# A phase I study in paediatric patients to evaluate the safety and pharmacokinetics of SPI-77, a liposome encapsulated formulation of cisplatin

**DOI:** 10.1054/bjoc.2001.1723

**Published:** 2001-04

**Authors:** G J Veal, M J Griffin, E Price, A Parry, G S Dick, M A Little, S M Yule, B Morland, E J Estlin, J P Hale, A D J Pearson, H Welbank, A V Boddy

**Affiliations:** Cancer Research Unit; Department of Child Health, Medical School, University of Newcastle-upon-Tyne, Newcastle-upon-Tyne, NE2 4HH, UK; Royal Marsden Hospital, Sutton, Surrey, SM2 5PT, UK; Yorkhill Hospital, Glasgow G3 8SJ, UK; Birmingham Childrens Hospital, Birmingham, B4 6NH, UK; Royal Hospital for Sick Children, Bristol, BS2 8BJ, UK; ALZA Corporation, Mountain View, CA, USA

**Keywords:** SPI-77, liposomal cisplatin, pharmacokinetics, paediatrics, phase I

## Abstract

Pre-clinical studies indicate that cisplatin encapsulated in STEALTH®liposomes (SPI-77) retains anti-tumour activity, but has a much reduced toxicity, compared to native cisplatin. A phase I study was conducted to determine the toxicity and pharmacokinetics of SPI-77 administered to children with advanced cancer not amenable to other treatment. Paediatric patients were treated at doses ranging from 40 to 320 mg m^−2^by intravenous infusion every 4 weeks. Blood samples taken during, and up to 3 weeks after, administration and plasma and ultrafiltrate were prepared immediately. Urine was collected, when possible, for 3 days after administration. SPI-77 administration was well tolerated with the major toxicity being an infusion reaction which responded to modification of the initial infusion rate of SPI-77. Limited haematological toxicity and no nephrotoxicity were observed. No responses to treatment were seen during the course of this phase I study. Measurement of total plasma platinum showed that cisplatin was retained in the circulation with a half life of up to 134 h, with maximum plasma concentrations approximately 100-fold higher than those reported following comparable doses of cisplatin. Comparison of plasma and whole blood indicated that cisplatin was retained in the liposomes and there was no free platinum measurable in the ultrafiltrate. Urine recovery was less than 4% of the dose administered over 72 h. Results from this phase I study indicate that high doses of liposomal cisplatin can safely be given to patients, but further studies are required to address the issue of reformulation of liposomally bound cisplatin. © 2001 Cancer Research Campaign http://www.bjcancer.com


British Journal of Cancer (2001) 84(8), 1029–1035
© 2001 Cancer Research Campaign
doi: 10.1054/ bjoc.2001.1723, available online at http://www.idealibrary.com on IDEAL®

http://www.bjcancer.com

### A phase I study in paediatric patients to evaluate the safety and pharmacokinetics of SPI-77, a liposome encapsulated formulation of cisplatin

**GJ Veal[1], MJ Griffin[1], E Price[2], A Parry[2], GS Dick[3], MA Little[3], SM Yule[4], B Morland[5], EJ Estlin[6], JP Hale[2], ADJ Pearson[2], H Welbank[7] and AV Boddy[1]**

[1]Cancer Research Unit and [2]Department of Child Health, Medical School, University of Newcastle-upon-Tyne, Newcastle-upon-Tyne NE2 4HH, UK; [3]Royal Marsden Hospital, Sutton, Surrey SM2 5PT, UK; [4]Yorkhill Hospital, Glasgow G3 8SJ, UK; [5]Birmingham Childrens Hospital, Birmingham B4 6NH, UK; [6]Royal Hospital for Sick Children, Bristol BS2 8BJ, UK; [7]ALZA Corporation, Mountain View, CA, USA

**Summary** Pre-clinical studies indicate that cisplatin encapsulated in STEALTH® liposomes (SPI-77) retains anti-tumour activity, but has a much reduced toxicity, compared to native cisplatin. A phase I study was conducted to determine the toxicity and pharmacokinetics of SPI-77 administered to children with advanced cancer not amenable to other treatment. Paediatric patients were treated at doses ranging from 40 to 320 mg $m^{-2}$ by intravenous infusion every 4 weeks. Blood samples taken during, and up to 3 weeks after, administration and plasma and ultrafiltrate were prepared immediately. Urine was collected, when possible, for 3 days after administration. SPI-77 administration was well tolerated with the major toxicity being an infusion reaction which responded to modification of the initial infusion rate of SPI-77. Limited haematological toxicity and no nephrotoxicity were observed. No responses to treatment were seen during the course of this phase I study. Measurement of total plasma platinum showed that cisplatin was retained in the circulation with a half life of up to 134 h, with maximum plasma concentrations approximately 100-fold higher than those reported following comparable doses of cisplatin. Comparison of plasma and whole blood indicated that cisplatin was retained in the liposomes and there was no free platinum measurable in the ultrafiltrate. Urine recovery was less than 4% of the dose administered over 72 h. Results from this phase I study indicate that high doses of liposomal cisplatin can safely be given to patients, but further studies are required to address the issue of reformulation of liposomally bound cisplatin. © 2001 Cancer Research Campaign http://www.bjcancer.com

**Keywords**: SPI-77; liposomal cisplatin; pharmacokinetics; paediatrics; phase I

Cisplatin (cis-diamminedichloroplatinum) was first introduced into clinical practice in 1971 and has now been licensed for use in the treatment of cancer for over 20 years. Despite the continued search for new platinum complexes and cisplatin analogues with increased efficacy over the parent compound, cisplatin remains the mainstay of treatment for a number of solid tumour types. Cisplatin is currently an essential component in many clinical paediatric protocols for the treatment of osteogenic sarcoma, neuroblastoma and central nervous system tumours (Bramwell et al, 1992; Pearson et al, 1992; Buckner et al, 1999). In addition, cisplatin has a pivotal role in the foundation of curative regimens in ovarian and testicular cancers and is also effective in the treatment of bladder, cervical, oesophageal and small cell lung cancers, amongst others.

The clinical application of cisplatin is predominantly limited by the considerable side effects of cisplatin treatment, which include acute toxicity such as nausea and vomiting and chronic side effects of nephrotoxicity, ototoxicity and neurotoxicity (Krakoff, 1979; Vermorken et al, 1983; Cavaletti et al, 1992). While the search for superior platinum drugs continues, it is essential that methods are investigated to optimize the current clinical use of cisplatin in both paediatric and adult patient populations. Although a variety of different approaches have been undertaken to limit its side effects, none have proven wholly successful in reducing either the acute or chronic toxicities associated with cisplatin therapy.

*Received 11 August 2000*
*Revised 11 November 2000*
*Accepted 22 January 2001*

*Correspondence to:* AV Boddy

Recent studies have shown that encapsulation of antineoplastic agents in liposomes can maintain antitumour activity whilst reducing drug toxicity (Rahman et al, 1990; Gill et al, 1995). Using this approach it is feasible that high plasma levels of free cisplatin, which are associated with the side effects of the drug, may be reduced whilst at the same time allowing more specific targeting of cisplatin to the tumour. This type of liposome delivery system has previously been used successfully for the delivery of doxorubicin in both animal and patient studies (Papahadjopoulous et al, 1991; Vaage et al, 1992; Uziely et al, 1995; Muggia et al, 1997).

SPI-77 (STEALTH® liposomal cisplatin) is a formulation of cisplatin encapsulated in sterically stabilized liposomes. These liposomes are thought to accumulate in tumours following extravasation through the tumour vasculature, due to their small size, long circulation time and reduced interaction with components of the blood including lipoproteins, cells, opsonins and other liposomes (Woodle and Lasic, 1992). Preclinical studies in tumour-bearing mice showed SPI-77 to have superior antitumour activity compared to the same cumulative dose of cisplatin with higher cumulative doses of SPI-77 being well tolerated and exhibiting increased antitumour affect (Newman et al, 1999). Significantly, SPI-77-treated animals had a 28-fold higher tumour exposure to platinum, as determined by area-under-the-curve (AUC), than cisplatin-treated animals, but a 4-fold lower exposure of platinum to the kidneys, which represents the primary target organ of toxicity associated with cisplatin treatment.

A phase I study of SPI-77 in children in the UK was conducted in parallel to the adult phase I study in the UK and Europe. This study was designed to determine the incidence of toxicity, maximum tolerated dose (MTD) and pharmacokinetics of SPI-77 when given every 4 weeks in paediatric patients with advanced cancer not amenable to other treatment.

### PATIENTS AND METHODS

### Patients

The study was performed between October 1997 and March 1999. Patients with different tumour types (Table 1) were recruited from 4 centres in the United Kingdom. Patients eligible for the study were aged 1–17 years with histologically confirmed malignancy for which no conventional therapy offered the possibility of cure or palliation. Previous chemotherapy must have ceased 4 weeks prior to recruitment to the study (6 weeks for nitrosourea) and 4 weeks must have elapsed since either cancer surgery or radiotherapy to measurable lesions. Further inclusion criteria were a Lansky performance status ≥ 30%, an adequate bone marrow function (absolute neutrophil count $\geq 1.0 \times 10^9$ $l^{-1}$, platelet count $\geq 100 \times 10^9$ $l^{-1}$), an adequate liver function (total bilirubin level within upper limit of normal, alanine aminotransferase and aspartate aminotransferase ≤ 1.5 upper limit of normal), an adequate renal function (glomerular filtration rate (GFR) > 50 ml $min^{-1}$ 1.73 $m^{-2}$) and no clinical evidence of hearing loss determined by audiometry (i.e., no toxicity ≥ grade 2 Common Toxicity Criteria (CTC)). Written informed consent was obtained from the patient's parent or legal guardian and consent was also sought from the patient where deemed appropriate.

Excluded from the study were patients with a life expectancy of less than 6 weeks, signs or symptoms of acute infection requiring systemic therapy, neurotoxicity from previous anti-cancer treatment or significant neuropathy (> grade 1 CTC) from any cause. Further exclusion criteria were a history of allergic reaction to cisplatin- or platinum-containing compounds, use of another investigational agent within 30 days of dosing with SPI-77 or prior treatment with SPI-77.

Pretreatment evaluation consisted of radiologic assessment of disease, ECG and audiogram within 14 days prior to the first dose of SPI-77. Further evaluation included a full medical history, physical examination, neurologic examination, an estimate of the GFR by $^{51}$Cr-EDTA plasma clearance, urine analysis, haematology including WBC with differential, red blood cell, haemoglobin, haematocrit and platelet count, and serum chemistry, glucose and a lipid profile. A urine pregnancy test was also carried out in female patients of childbearing potential. The study was approved by the appropriate Local Research Ethics Committee (LREC).

### Drug formulation

SPI-77 was supplied by SEQUUS Pharmaceuticals, Inc (Brentford, UK) in single-dose sterile vials as an isotonic suspension containing a cisplatin concentration of 1 mg $ml^{-1}$. The total lipid content of the SPI-77 formulation was 71 mg $ml^{-1}$ (hydrogenated soy phosphatidylcholine, cholesterol and the polymer MPEG-DSPE at an approximate 51:44:5 molar ratio), giving a drug-to-lipid ratio of approximately 14 μg cisplatin per 1 mg of lipid. The formulation also contained 10% sucrose, 1 mM sodium chloride and 10 mM histidine at pH 6.5. The mean liposome particle diameter was 110 nm and the cisplatin encapsulation exceeded 90%. Prior to infusion, SPI-77 was diluted to a final concentration of 0.2 mg $ml^{-1}$ in normal saline or D5W (dextrose 5% in water). All doses of SPI-77 quoted in this study are given in cisplatin equivalents of mg $m^{-2}$.

### Treatment plan

Patients received SPI-77 every 4 weeks for a total of 6 doses or until disease progression, whichever occurred earlier. 3 patients were initially enrolled for each dose level. SPI-77 was administered by intravenous (i.v.) infusion at a rate of 10 mg $m^{-2}$ $h^{-1}$ for the first 15 minutes, which increased to 40 mg $m^{-2}h^{-1}$ for the remainder of the infusion, assuming no adverse reaction had occurred. All SPI-77 infusions were administered using a paediatric approved infusion pump. All patients received premedication with dexamethasone (0.15 mg $kg^{-1}$ qds oral or i.v.), chlorpheniramine (0.05 mg $kg^{-1}$ qds oral or i.v.) and ranitidine (3 mg $kg^{-1}$ bd oral or 1 mg $kg^{-1}$ i.v. tds) starting 24 h prior to SPI-77 infusion and continuing for 24 h after the infusion. The starting dose of SPI-77 was 40 mg $m^{-2}$ for the first 3 patients with planned dose escalations as follows: 80, 120, 200, 320, 520, 840 mg $m^{-2}$, provided that no dose-limiting toxicities were observed. The last patient at each dose level was observed for at least 4 weeks before the first patient at the subsequent dose level was treated. If a dose-limiting toxicity (DLT) occurred in one of 3 patients at any given dose level, then 3 additional patients were treated at that level. The maximum tolerated dose (MTD) was defined as the dose immediately below that at which 2 out of 6 patients experienced a DLT.

DLT was defined as NCI CTC grade 3 thrombocytopenia or neutropenia lasting more than 7 days, grade 3 hepatic toxicity which does not resolve at least to grade 1 prior to the end of the cycle or any grade 3 or higher neurological or kidney/bladder toxicity. Any other grade 3 or 4 event, with the exception of nausea, vomiting, alopecia, weight change, fatigue and allergic reactions was also considered dose limiting.

### Clinical evaluations

During each 4 week cycle of SPI-77 treatment, haematology and serum chemistry and electrolytes were checked on a weekly basis. Urine analysis and neurologic examination were performed at the end of each cycle and an audiogram and GFR measurement carried out by $^{51}$Cr-EDTA plasma clearance at the end of cycles 2, 4 and 6. Upon completion of treatment, an electrocardiogram was also performed. Tumour evaluation and assessment of response was carried out at the end of cycles 2, 4 and 6 or at early withdrawal for patients not completing the entire course of treatment.

### Pharmacokinetic sampling

During the first course of treatment, whole blood samples (approx. 3 ml) were collected in heparinized tubes prior to administration of SPI-77, hourly during infusion and at the end of infusion. Further samples were taken at 1, 3, 5, 24, 96, 120 and 168 hours post infusion and weekly thereafter for 2 additional weeks. For subsequent

© 2001 Cancer Research Campaign courses at the same dose level, samples were taken at pre-dosing, hourly during infusion and at the end of infusion. A 400 µl aliquot of blood was removed from each sample and the remainder centrifuged for 10 minutes at 4˚C (2000 ***g***) to obtain the plasma fraction. Whole blood and plasma samples (400 µl) were diluted 10-fold with 0.1% triton-X-100 +0.2% nitric acid to a final volume of 4 ml. These samples were stored at –20˚C prior to analysis. The remaining plasma was centrifuged for 30 minutes at 1500 ***g*** using Amicon Centrifree ultrafiltration devices to obtain a minimum of 200 µl of plasma ultrafiltrate and the samples stored at –20˚C prior to analysis.

Urine samples were collected, where possible, beginning on the first day of treatment with the initiation of dosing and continuing each day for 3 consecutive days. 24 h pooled urine volumes were recorded and 10 ml of each pooled urine sample stored for analysis along with a 10 ml predose spot urine sample.

All platinum analyses were carried out at the Cancer Research Unit, Newcastle, by flameless atomic absorption spectrophotometry using a Perkin-Elmer AAnalyst 600 graphite furnace spectrometer (Perkin-Elmer Ltd, Beaconsfield, UK). Sample analysis was carried out using a 4-stage heating programme with temperatures ramping from 80 to 2800˚C. Total platinum concentrations were determined in whole blood and plasma samples and free platinum levels determined in plasma ultrafiltrates (Peng et al, 1997). All samples were analysed in duplicate and the intra- and interassay coefficients of variation for a quality assurance sample had to be <10% for an assay to be valid. The limit of detection for the AAS was 0.10 µg ml$^{-1}$.

### Data interpretation

The total and free platinum pharmacokinetic parameters in plasma and urine were calculated by standard non-compartmental analysis with actual sampling times used in the calculations. Analysis of all pharmacokinetic data was performed using WinNonlin.

### RESULTS

### Patients and treatment

A total of 18 patients were recruited to the study. Patient characteristics are shown in Table 1. 3 patients were treated at the starting dose of 40 mg m$^{-2}$ (4 courses in total) and 4 patients were treated at 80 mg m$^{-2}$ (4 courses) due to the withdrawal of 1 patient following a grade 3 infusion reaction. 3 patients were treated at 120 mg m$^{-2}$ (6 courses), 4 patients at 200 mg m$^{-2}$ (6 courses) and 3 patients at 320 mg m$^{-2}$ (5 courses) giving a total of 25 courses administered overall. 10 patients received a single course of treatment, 4 patients received 2 courses (at doses of 40, 80, 120 and 320 mg m$^{-2}$) and 2 patients received 3 courses (doses of 120 and 200 mg m$^{-2}$). No further dose escalations were carried out due to concerns regarding the high doses of cisplatin being administered to patients in both the adult (Schellens et al, 1998) and paediatric phase I studies and the lack of detectable levels of free platinum measured in patient ultrafiltrate samples, indicating that the cisplatin was being retained within the liposomes. Following the occurrence of infusion reactions in 3 patients receiving doses of SPI-77 of 40 and 80 mg m$^{-2}$, in addition to similar adverse infusion-related reactions in the parallel phase I adult study, the initial infusion rate was decreased to 1 mg m$^{-2}$ h$^{-1}$ for the first 15 minutes, followed by a step increase up to 40 mg m$^{-2}$ h$^{-1}$ for the remainder of the infusion, giving an increased duration of infusion at doses of 120 mg m$^{-2}$ and above. Due to this increased duration of infusion, the final concentration of SPI-77 was increased from 0.2 mg ml$^{-1}$ to 0.3 mg ml$^{-1}$, in order to maintain the total infusion volumes at acceptable levels.

**Table 1** Patient characteristics

| | |
|---|---|
| Number of patients | 18 |
| Male/female | 10/8 |
| Median age (range) | 10 (1–16) |
| Dose level (number of courses) | |
| 40 mg m$^{-2}$ | 3 (4) |
| 80 mg m$^{-2}$ | 4 (4) |
| 120 mg m$^{-2}$ | 3 (6) |
| 200 mg m$^{-2}$ | 4 (6) |
| 320 mg m$^{-2}$ | 3 (5) |
| Diagnosis | |
| Neuroblastoma | 5 |
| Rhabdomyosarcoma | 5 |
| PNET (intra/extracranial) | 2 (1/1) |
| Astrocytoma | 1 |
| Hepatocellular carcinoma | 1 |
| Ewings sarcoma | 1 |
| Small cell osteosarcoma | 1 |
| Medulloblastoma | 1 |

Of the 18 patients recruited to the study, 1 patient did not receive SPI-77 due to disease progression prior to treatment, 1 patient was withdrawn following a serious infusion reaction and 3 patients died after the first course of therapy due to disease progression. No responses to treatment were observed in any of the patients studied. The remaining 13 patients went off-study following 1 (6 patients), 2 (5 patients) and 3 (2 patients) courses of SPI-77 treatment due to disease progression. Of the 5 patients who received 2 courses of treatment, 3 had rhabdomyosarcoma, 1 neuroblastoma and 1 PNET. Of the 2 patients who received 3 courses, 1 had hepatocellular carcinoma and the other neuroblastoma.

### Toxicity

17 patients were evaluable for toxicity. Table 2 shows a summary of all the toxicities above CTC grade 1 recorded during this study. The most serious adverse event was a CTC grade 3 infusion reaction observed in 3 patients at doses of 40 and 80 mg m$^{-2}$ during the first course of treatment in each case. This led to 1 patient being withdrawn from the study and resulted in modification of the initial infusion rate of SPI-77 for further dose escalation as described above. The other 2 patients were able to recommence administration on the same day, following resolution of the symptoms and treatment with antihistamines, steroids and oxygen. No subsequent infusion reactions were seen in patients treated under the reviewed guidelines for initial SPI-77 infusion. Gastrointestinal toxicities were the most commonly observed in children receiving SPI-77, with a total of 65% of patients (11/17) experiencing vomiting during the course of treatment and a further 18% of patients (3/17) experiencing nausea. Neither of these toxicities exceeded CTC grade 2 in any of the patients studied. 1 patient experienced a CTC grade 4 GI bleed, but this was not thought to be treatment related. Haematological toxicity was limited to grade 2/3 anaemia in 35% of patients (6/17) and grade 2 thrombocytopenia in 12% of patients (2/17). In 1 of these patients bone marrow disease is likely to have compromised platelet levels. Other toxicities observed included grade 3 hypertension (1/17), grade 1 fever (3/17), grade 1/2 allergic reaction (4/17) and grade 1 hyponatrenia

© 2001 Cancer Research Campaign

**Table 2** Patient toxicities

| Toxicity | Grade | Number of patients (%) | Number of courses (%) |
|---|---|---|---|
| Gastrointestinal | | | |
| Vomiting | 1.2 | 11 (64) | 13 (52) |
| Nausea | 1.2 | 4 (24) | 4 (16) |
| GI bleed | 4[a] | 1 (6) | 1 (4) |
| Haematologic | | | |
| Anaemia | 2 | 4 (24) | 5 (20) |
| | 3[b] | 2 (12) | 3 (12) |
| Thrombocytopenia | 2 | 2 (12) | 2 (8) |
| Infusion reaction | 3[c] | 3 (18) | 3 (12) |
| Dermatologic | | | |
| Allergy/rash | 1.2 | 4 (24) | 4 (16) |
| Alopecia | 1 | 1 (6) | 1 (4) |
| Other toxicities | | | |
| Fever | 1 | 3 (18) | 3 (12) |
| Hypertension | 3[d] | 1 (6) | 1 (4) |

[a]Dose level 120 mg $m^{-2}$; [b]Dose levels 40 and 120 mg $m^{-2}$; [c]Dose levels 40 and 80 mg $m^{-2}$; [d]Dose level 80 mg $m^{-2}$.

(2/17). Altered serum chemistry/electrolytes such as hypokalaemia, hyperbilirubinaemia and decreased phosphate were never greater than grade 1 and were never reported in more than 6% of patients (1/17).

### Monitoring of blood lipids

Levels of cholesterol, HDL and LDL fractions and triglycerides were monitored during treatment with SPI-77 due to the high lipid content of the STEALTH® liposomes being administered. Peak cholesterol levels were observed at 8 days following administration of SPI-77 and then declined to levels approximating those seen prior to treatment by day 29. The relationship between dose of SPI-77 and corresponding increase in cholesterol levels is shown in Figure 1. Percentage increases in mean cholesterol levels at 8 days following SPI-77 administration were 77%, 102% and 244% at doses of 120, 200 and 320 mg $m^{-2}$ respectively. Similar increases were seen in levels of HDL and LDL, for example at a SPI-77 dose of 320 mg $m^{-2}$. Mean percentage increases from pretreatment levels of HDL and LDL measured at 8 days were 300% and 143% respectively. Variations in triglyceride levels were less clearly defined, but increased levels were seen in the majority of patients over a 28 day period following treatment with SPI-77.

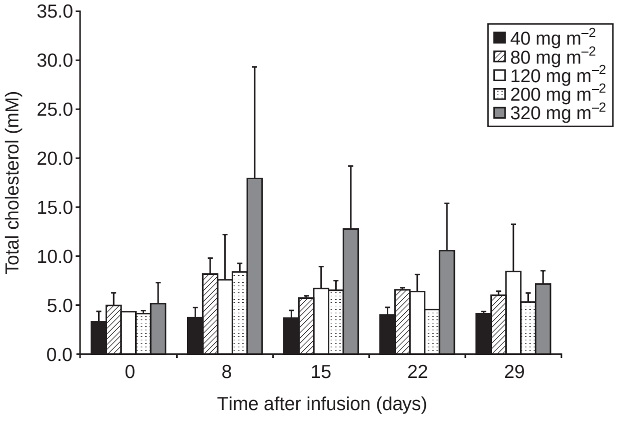

**Figure 1** Mean plasma levels of total cholesterol following SPI-77 administration at doses of 40 to 320 mg $m^{-2}$ following the first course of treatment. Error bars indicate standard error of the mean

### Pharmacokinetics

Blood samples were taken for analysis of platinum during infusion of SPI-77 and for up to 3 weeks after administration. These platinum concentration data were used to derive pharmacokinetic parameters for total platinum in plasma and whole blood. Full pharmacokinetic data were obtained for 12 patients and are shown in Table 3. Despite the high concentrations of cisplatin administered to patients in this study, concentrations of free platinum measured in the plasma ultrafiltrate remained below the limit of detection (0.10 μg $ml^{-1}$) throughout the treatment period. Cisplatin was detectable in plasma and whole blood after SPI-77 administration in all patients. Individual patient plasma time profiles representative of the dose levels studied are shown in Figure 2. At the highest dose level, plasma platinum concentrations were still measurable prior to administration of the second course of treatment, i.e. 4 weeks after drug infusion. A plot of total platinum area under the plasma concentration–time curve (AUC) versus the dose of SPI-77 is shown in Figure 3. The half-life values for total platinum in plasma, determined following SPI-77 treatment, were within the range of 38 to 134 h in all but 1 patient, indicating an approximate 3-fold variation. Distribution of cisplatin within whole blood was determined by the blood/plasma ratio and ranged from approximately 0.5–0.8 in the majority of patients studied (Table 3), this generally reflected the haematocrit in these patients. The pharmacokinetic profile of patient 2 was notably different from that of the other patients studied, with a significantly greater clearance, reduced AUC and half-life and a blood/plasma ratio approaching 1. Patient 8 received multiple blood transfusions within 72 hours of SPI-77 administration, which resulted in an increased rate of elimination and disruption of whole blood distribution (Figure 4). Urine data was obtained from 6 patients and indicated prolonged and limited excretion, with a value of 3.8 ± 0.9% (mean ± SD) of the dose excreted in the first 72 hours.

 *© 2001 Cancer Research Campaign*

**Table 3** Pharmacokinetics of total platinum in plasma following SPI-77 treatment

| Patient | Dose (mg m$^{-2}$) | Infusion time (h) | $C_{max}$ (µg ml$^{-1}$) | AUC (mg ml$^{-1}$ min) | Cl (ml h$^{-1}$) | $t_{1/2}$ (h) | Blood/plasma[a] |
|---|---|---|---|---|---|---|---|
| 1 | 40 | 2 | 34 | 145 | 20 | 38 | 0.72–0.88 |
| 2 | 40 | 4 | 8 | 4 | 255 | 11 | 0.69–1.00 |
| 3 | 40 | 5 | 37 | 181 | 17 | 65 | 0.54–0.66 |
| 5 | 80 | 3 | 71 | 402 | 5 | 56 | 0.49–0.80 |
| 7 | 80 | 5 | 103 | 818 | 13 | 134 | 0.60–0.88 |
| 8 | 120 | 4 | 88 | 442 | 18 | 41 | 0.31–0.79 |
| 9 | 120 | 3 | 111 | 679 | 19 | 86 | 0.57–0.85 |
| 10 | 120 | 4 | 151 | 1359 | 7 | 96 | 0.30–0.68 |
| 12 | 200 | 6 | 142 | 1412 | 9 | 78 | 0.55–0.76 |
| 13 | 200 | 6 | 177 | 1788 | 4 | 82 | 0.53–0.75 |
| 16 | 320 | 7 | 411 | 8027 | 2 | 132 | 0.54–0.73 |
| 18 | 320 | 6 | 187 | 2157 | 8 | 73 | 0.74–1.05 |

[a]Blood/plasma ratio given as a range determined over the 3 week sampling period.

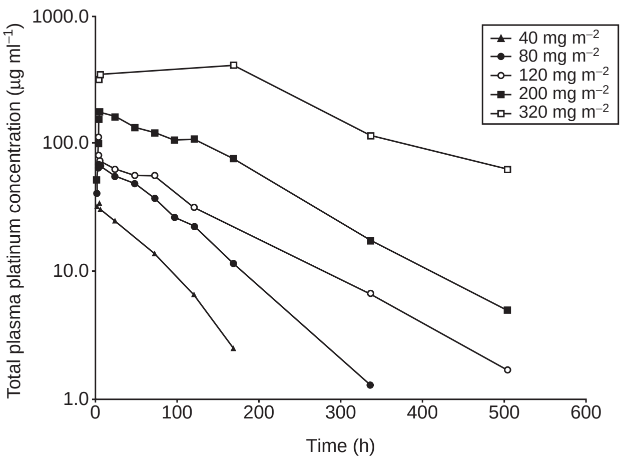

**Figure 2** Plasma concentrations of total platinum following SPI-77 administration at doses of 40 to 320 mg m$^{-2}$. Data show individual patient data representative of each dose level of SPI-77

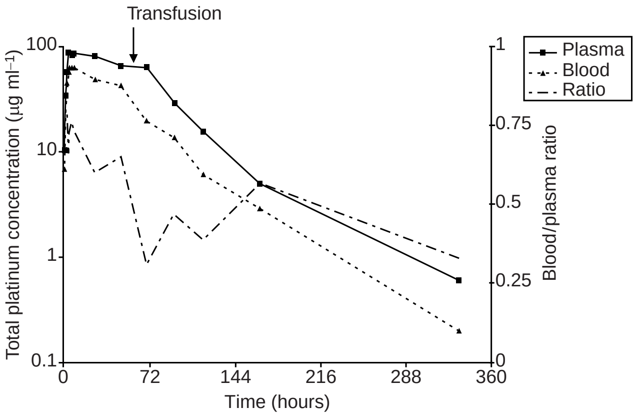

**Figure 4** Concentrations of total platinum in plasma and whole blood following SPI-77 administration at a dose of 120 mg m$^{-2}$ following the first course of treatment for patient 8. Patient received multiple blood transfusions within 72 h of SPI-77 administration

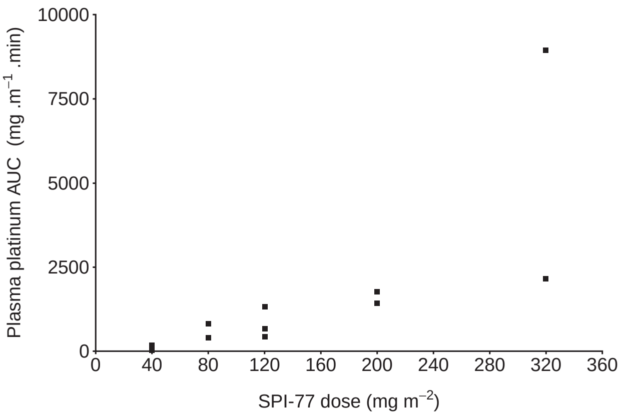

**Figure 3** Relationship between total platinum area under the plasma concentration–time curve (AUC) and the dose of SPI-77 administered

### DISCUSSION

The potential use of liposomes to deliver drugs to target sites of disease is a therapeutic approach that has been investigated with many different agents over the past 2 decades. Recent successes in this area of clinical research include the efficacy of liposomal doxorubicin in AIDS-related Kaposi's sarcoma and patients with refractory ovarian cancer (Muggia et al, 1997; Northfelt et al, 1997). Although the precise mechanisms by which liposomes selectively enter tumours and release drug are not completely understood, their successful application with doxorubicin has led to a resurgence of interest in this area. As liposome formulation issues and potential problems such as liposome stability and removal by elements of the immune system have been overcome with the STEALTH® liposomal system, the potential exists for improved delivery of a growing number of drugs for the treatment of many different types of disease. In addition, results from previous clinical trials give an indication as to how patients are likely to respond to the administration of STEALTH® liposomes

*© 2001 Cancer Research Campaign* 

and the potential toxicities that may be associated with their use. Therefore the main issue regarding their use for the delivery of other therapeutic agents will largely be concerned with obtaining the desired balance between encapsulation and release of the drug of interest.

Preclinical results with SPI-77, a formulation of cisplatin encapsulated in STEALTH® liposomes, showed promising results in comparison with native cisplatin. Data from tumour-bearing mouse models showed a significantly greater antitumour activity compared to cisplatin and a significantly increased tumour exposure to platinum. This coincided with a decreased platinum exposure to the kidneys, the principal organ of cisplatin toxicity (Newman et al, 1999). On the basis of these preclinical results, in addition to the previous success of STEALTH® liposomal doxorubicin, phase I studies in both adult and paediatric patients, with advanced cancer not amenable to other cancer treatments, were performed in the UK and Europe. These studies were designed to determine the incidence of toxicity, MTD and pharmacokinetics of SPI-77 in these 2 separate patient populations. The rate of patient recruitment to the 2 studies was such that the adult phase I study was always one dose level ahead of the paediatric study, i.e. children were not enrolled for the next dose level until the specified number of adult patients had been treated at that dose.

Complete pharmacokinetic profiles were obtained from 12 of the patients taking part in this study. Cisplatin levels were determined in 2 different forms, total cisplatin concentrations were determined in both patient plasma and whole blood samples and concentrations of free platinum were determined in plasma ultrafiltrate samples. The cisplatin blood/plasma concentration ratio following SPI-77 treatment reflected the haematocrit in most patients. This contrasts with the equal distribution of platinum between plasma and red cells following native cisplatin administration (Manaka and Wolf, 1978). This information, in combination with the fact that there was no measurable free/active drug in plasma ultrafiltrate samples strongly suggests that the drug was retained within the liposomes following administration.

Plasma concentrations of total platinum following treatment with SPI-77 were considerably higher than those reported following administration of native cisplatin in children (Bues-Charbit et al, 1987; Dominici et al, 1989). For example, the total platinum AUC values obtained at a SPI-77 dose of 80 mg $m^{-2}$ in this phase I study were approximately 100-fold higher than the total platinum AUC values previously reported following an equivalent dose of cisplatin (Pratt et al, 1981). This marked increase in total platinum AUC can largely be explained by the apparent decrease in clearance of total platinum and the low percentage of total platinum dose excreted in the urine of patients following SPI-77 administration. Total platinum clearance values observed for SPI-77 in the current study were ≤20 ml $h^{-1}$ in all but 1 patient, approximately 100- to 1000-fold lower than clearance values of 10–20 l $h^{-1}$ $m^{-2}$ commonly reported for cisplatin (Bues-Charbit et al, 1987; Peng et al, 1997). Similarly, the limited excretion of cisplatin in the urine of children observed following SPI-77 administration, with approximately 4% of the administered dose excreted in the first 72 hours, was significantly less than seen with native cisplatin, with reported values of 27–35% of the dose excreted in 48 hours (Bues-Charbit et al, 1987; Peng et al, 1997). Overall, the half-life of cisplatin was prolonged following SPI-77 administration, but was not dissimilar to that of total plasma platinum reported following cisplatin administration in previous clinical studies (Pratt et al, 1981; Dominici et al, 1989). There was some evidence of non-linearity, with lower clearance and longer half-life of drug at the higher dose levels. This was also apparent from individual patient plasma time profiles representative of the dose levels studied (Figure 2).

One patient (patient 2) showed a different pharmacokinetic profile to the other patients with a blood/plasma ratio approaching unity and a much shorter half-life due to a higher clearance of cisplatin. As the data suggest that the drug was normally retained in the liposomes following administration of SPI-77, it seems likely that the different pharmacokinetics observed in this patient was due to altered handling of the liposomes. This may involve a decrease in liposome stability through interaction with plasma proteins or an increased clearance of liposome from the plasma. It is interesting to note that no increase in cholesterol levels was seen at 8 days after infusion of SPI-77 in this patient as compared to increases of 7 and 46% from pretreatment levels in the other 2 patients treated at the same dose level. The only other patient who exhibited a significantly different pharmacokinetic profile was patient 8 who received multiple blood transfusions within 72 hours of SPI-77 administration. This resulted in an increased rate of elimination and disruption of whole blood distribution (Figure 4).

The most striking clinical or biochemical observation was hyperlipidaemia with percentage increases in mean cholesterol levels at 8 days following administration being 77%, 102% and 244% at doses of 120, 200 and 320 mg $m^{-2}$ respectively. Similar increases were seen in levels of HDL and LDL. Despite the fact that cholesterol levels returned to normal before the scheduled retreatment time, the apparent exaggeration of this adverse effect seen at a SPI-77 dose of 320 mg $m^{-2}$ raises potential concerns with regards to further dose escalation. Other toxicities, including antiproliferative effects, were generally mild (CTC grade 1/2) with the only serious adverse event being a GI bleed which was not thought to be treatment related, due to a lack of related side effects and the length of time after administration that the toxicity occurred. Overall, administration of SPI-77 was well tolerated in paediatric patients with doses 3-fold higher than would be achievable with native cisplatin. However, despite this increase in cisplatin dosage and the high levels of total platinum in plasma, no responses were seen in any of the patients studied.

In summary, the data from this study indicate that the encapsulation of cisplatin in STEALTH® liposomes results in an altered toxicity profile and significantly different pharmacokinetics as compared with standard cisplatin administration in paediatric patients. This is in agreement with preliminary data obtained in a parallel phase I study of SPI-77 in adults (Schellens et al, 1998). The absence of nephrotoxicity following treatment with SPI-77 may be explained predominantly by the lack of detectable free platinum in the plasma, but may also be connected with the significantly decreased percentage of platinum excreted in the urine compared with cisplatin treatment. The major abnormal finding in the study was hypercholesterolaemia with low-grade anaemia and thrombocytopenia being the only observed antiproliferative effects. The pharmacokinetics of SPI-77 were significantly altered from those associated with cisplatin treatment and implied that the cisplatin was maintained inside the liposome with no detectable free cisplatin measured in plasma at any point following administration. Although preclinical data suggested that low plasma levels of free cisplatin would explain the lack of toxicity seen with SPI-77 in murine tumour models, the complete retention of cisplatin within the liposomes was not predicted as significant antitumour activity was observed (Newman et al, 1999). Both the paediatric

 © 2001 Cancer Research Campaign and adult phase I studies were stopped at a dose of 320 mg $m^{-2}$ in order to address the issue of reformulation of liposomally bound cisplatin, i.e. to attempt to obtain an optimal balance between encapsulation and release of cisplatin. Despite the potential problems with formulation of liposomally bound cisplatin highlighted in this study, the limited toxicity reported in patients in the study does show that high doses of liposomal cisplatin can safely be given to patients. This would suggest that if the product can be reformulated to obtain the desired balance between encapsulation and release, this approach has the potential to lead to an improved method of administering cisplatin in a clinical setting.

### REFERENCES

Bramwell VH, Burgers M, Sneath R, Souhami R, van Oosterom AT, Voûte PA, Rouesse J, Spooner D, Craft AW, Somers R, Pringle J, Malcolm AJ, van der Eijken J, Thomas D, Uscinska B, Machin D and van Glabbeke M (1992) A comparison of two short intensive adjuvant chemotherapy regimens in operable osteosarcoma of the limbs in children and young adults: the first study of the European Osteosarcoma Intergroup. *J Clin Oncol* **10**: 1579–1531

Buckner JC, Peethambaram PP, Smithson WA, Groover RV, Schomberg PJ, Kimmel DW, Raffel C, OFallon JR, Neglia J and Shaw EG (1999) Phase II trial of primary chemotherapy followed by reduced-dose radiation for CNS germ cell tumors. *J Clin Oncol* **17**: 933–940

Bues-Charbit M, Gentet JC, Bernard JL, Breant V, Cano JP and Raybaud C (1987) Continuous infusion of high-dose cisplatin in children: pharmacokinetics of free and total platinum. *Eur J Cancer Clin Oncol* **23**: 1649–1652

Cavaletti G, Marzorati L, Bogliun G, Colombo N, Marzola M, Pittelli MR and Tredici G (1992) Cisplatin-induced peripheral neurotoxicity is dependent on total dose-intensity and single dose-intensity. *Cancer* **69**: 203–207

Dominici C, Petrucci F, Caroli S, Alimonti A, Clerico A and Castello MA (1989) A pharmacokinetic study of high-dose continuous infusion cisplatin in children with solid tumors. *J Clin Oncol* **7**: 100–107

Gill PS, Espina BM, Muggia F, Cabriales S, Tulpule A, Esplin JA, Liebman HA, Forssen E, Ross ME and Levine AM (1995) Phase I/II clinical and pharmacological evaluation of liposomal daunorubicin. *J Clin Oncol* **13**: 996–1003

Krakoff IH (1979) Nephrotoxicity of cis-dichlorodiammineplatinum (II). *Cancer Treat Rep* **63**: 1523–1525

Manaka RC and Wolf W (1978) Distribution of *cis*-platin in blood. *Chem Biol Interactions* **22**: 353–358

Muggia FM, Hainsworth JD, Jeffers S, Miller P, Groshen S, Tan M, Roman L, Uziely B, Muderspach L, Garcia A, Burnett A, Greco FA, Morrow CP, Paradiso LJ and Liang LJ (1997) Phase II study of liposomal doxorubicin in refractory ovarian cancer: antitumor activity and toxicity modification by liposomal encapsulation. *J Clin Oncol* **15**: 987–993

Newman MS, Colbern GT, Working PK, Engbers C and Amantia MA (1999) Comparative pharmacokinetics, tissue distribution, and therapeutic effectiveness of cisplatin encapsulated in long-circulating, pegylated liposomes (SPI-077) in tumor-bearing mice. *Cancer Chemother Pharmacol* **43**: 1–7

Northfelt DW, Dezube BJ, Thommes JA, Levine R, Von Roenn JH, Dosik GM, Rios A, Krown SE, DuMond C and Mamelok RD (1997) Efficacy of pegylated-liposomal doxorubicin in the treatment of AIDS-related Kaposi's sarcoma after failure of standard chemotherapy. *J Clin Oncol* **15**: 653–659

Papahadjopoulous D, Allen TN, Gabizon A, Mayhew E, Matthay K, Huang SK, Lee KD, Woodle MC, Lasic DD, Redemann C and Martin FJ (1991) Sterically stabilized liposomes: Improvement in pharmacokinetics and antitumor therapeutic efficacy. *Proc Natl Acad Sci (USA)* **88**: 11460–11464

Pearson AD, Craft AW, Pinkerton CR, Meller ST and Reid MM (1992) High-dose rapid schedule chemotherapy for disseminated neuroblastoma. *Eur J Cancer* **28A**: 1654–1659

Peng B, English M, Boddy AV, Price L, Wyllie R, Pearson ADJ, Tilby MJ and Newell DR (1997) Cisplatin pharmacokinetics in children. *Eur J Cancer* **33**: 1823–1828

Pratt CB, Hayes A, Green AA, Evans WE, Senzer N, Howarth CB, Ransom JL and Crom W (1981) Pharmacokinetic evaluation of cisplatin in children with malignant solid tumors: a phase II study. *Cancer Treat Rep* **65**: 1021–1026

Rahman A, Treat J, Roh JK, Potkul LA, Alvord WG, Forst D and Woolley PV (1990) A phase I clinical trial and pharmacokinetic evaluation of liposome encapsualted doxorubicin. *J Clin Oncol* **8**: 1093–1100

Schellens JHM, Meerum Terwogt J, Groenewegen G, Blijham GH, Ten Bokkel Huinink WW, Smart M, Maliepaard M. Floot B, Welbank H and Beijnen JH (1998) Phase I and pharmacologic study of SPI-77, a novel stealth liposomal encapsulated formulation of cisplatin (CDDP). *Eighty-ninth annual meeting, Proceedings American Association Cancer Research* **39**: P2218

Uziely B, Jeffers S, Isacson R, Kutsch K, Wei-Tsao D, Yehoshua Z, Libson E, Muggia FM and Gabizon A (1995) Liposomal doxorubicin (Dox-SL™): Antitumor activity and unique toxicities during two complementary phase I studies. *J Clin Oncol* **13**: 1777–1785

Vaage J, Mayhew E, Lasic D and Martin F (1992) Therapy of primary and metastatic mouse mammary carcinoma with doxorubicin encapsulated in long circulating liposomes. *Int J Cancer* **51**: 942–948

Vermorken JB, Kaptein TS, Hart AAM and Pinedo HM (1983) Ototoxicity of cisdiamminedichloroplatinum (II). Influence of dose, schedule and mode of administration. *Eur J Cancer Clin Oncol* **19**: 53–58

Woodle MC and Lasic DD (1992) Sterically stabilized liposomes. *Biochem Biophys Acta* **1113**: 171

*© 2001 Cancer Research Campaign*
